# Interference-Free Determination of Trace Copper in Freshly Ripened Honeys by Flame Atomic Absorption Spectrometry Following a Preconcentration by Solid-Phase Extraction and a Two-Step Elution Process

**DOI:** 10.1007/s00244-013-9961-x

**Published:** 2013-10-29

**Authors:** Pawel Pohl, Helena Stecka, Piotr Jamroz

**Affiliations:** Division of Analytical Chemistry, Faculty of Chemistry, Wroclaw University of Technology, 50-370 Wrocław, Poland

## Abstract

A fast and straightforward procedure aimed at separating copper (Cu) ions from monosacharides and preconcentrating their traces before flame atomic absorption spectrometry (FAAS) measurements was developed, and its suitability was evaluated by the analysis of freshly ripened honeys on the content of this environmentally and physiologically relevant element. This procedure included the passage (at 20 mL/min) of 10 % (m/v) solutions of honeys (100 mL) through resin beds of Dowex 50 W × 8-400 to retain Cu by solid-phase extraction (SPE) and separate it from the glucose and fructose matrix. In turn, SPE columns were rinsed at 20 mL/min with 20 mL of water and subsequently washed with 20 mL of a 0.5 mol/L HNO_3_ solution (at 2.0 mL/min) to elute potassium and sodium. Preconcentrated Cu was stripped (at 2.0 mL/min) with 5.0 mL of a 2.0 mol/L HCl solution and determined by FAAS. The proposed procedure was used for the analysis of six ripened monoflower and multiflower honeys, enabling the measurement of Cu within the range of 0.17–0.42 μg/g and with a precision of 3–10 %. Recoveries of Cu added to respective honey solutions were within 94–102 %, proving the good accuracy of this procedure. The detection limit of Cu achieved with this SPE preconcentration/separation procedure and FAAS detection was 3.6 ng/g.

Ripened honeys exhibit a great variability in the content of copper (Cu) mostly due to (1) their floral and regional variations and (2) the different kinds of environmental pollutions that contaminate them (Bogdanov 2006; Pohl 2009). This can be seen from Table 1, where the data for honeys from different countries are listed and vary from levels lower than the detection limit to 34 μg/g; however, most commonly the concentration of Cu is approximately 1 μg/g. An additional source of contamination of honey with Cu could be beekeeping practices and honey processing after harvesting (Pohl 2009). Steel and galvanized tools and containers used for the honey harvesting, ripening, and packing quite commonly undergo corrosion in contact with the honey, and this results in the release of simple ions of Cu and other related elements (chromium, iron [Fe], nickel) (Paramas et al. 2000).

**Table 1:** Concentration ranges of Cu measured in monofloral and multifloral honeys of the different origin measured using FAAS

Country	Concentration (μg/g)	Reference
Argentina	ND	Baroni et al. (2009)
Brazil	ND–33.77	dos Santos et al. (2008)
Czech	0.11–1.50	Vorlova and Celechovska (2002), Lachman et al. (2007)
India	1.29–2.90	Nanda et al. (2003, 2009)
Pakistan	0.12–0.91	Khan et al. (2006)
Poland	ND–1.82	Wieczorek et al. (2006), Madejczyk and Baralkiewicz (2008), Juszczak et al. (2009), Pohl and Sergiel (2010, 2012), Grembecka and Szefer (2013)
Saudi Arabia	0.21–0.39	Osman et al. (2007)
Spain	0.04–1.73	Latorre et al. (1999); Hernandez et al. (2005)
Turkey	ND–3.50	Uren et al. (1998); Yilmaz and Yavuz 1999; Erbilir and Erdogrul 2005; Turhan (2007)

*ND* not detected

The intake of high amounts of Cu with dietary products can be manifested by various malfunctions in the nervous system and the formation of reactive oxygen species, which lead to severe oxidative damages and dysfunctions of almost all biological cell molecules, including lipids, proteins, and nucleic acids (Bremner 1998; Gaetke and Chow 2003). Given the necessity of a fast and straightforward evaluation of honey safety and quality in reference to its contents of toxic trace Cu and other related elements, it could be reasonably argued that analytical methods enabling such reliable determinations in honey would be highly important and desirable.

Flame atomic absorption spectrometry (FAAS) with an air-acetylene flame is a well-established element specific detection technique commonly used for the analysis of honey as to the content of Cu and other trace elements (Pohl 2009; Pohl et al. 2009). Unfortunately, organic (glucose and fructose mostly) and inorganic (cationic and anionic minerals, such as chlorine, potassium [K], sodium [Na], and phosphorous) constituents of the honey matrix are often responsible for different kind of spectral and nonspectral interferences that accompany FAAS measurements (Hernandez et al. 2005; dos Santos et al. 2008; Pohl et al. 2012a, b). For that reason, analyzed samples of honey are mineralized before determinations by FAAS to simplify the sample matrix and eliminate related matrix effects. Apparently, from the literature, honeys are dry ashed in a number of cases (Uren et al. 1998; Latorre et al. 1999; Yilmaz and Yavuz 1999; Vorlova and Celechovska 2002; Erbilir and Erdogrul 2005; Wieczorek et al. 2006; Lachman et al. 2007; Osman et al. 2007) by high-temperature incinerations of relatively high sample masses (5–20 g). The resulting ashes are evaporated in the presence of added HCl or HNO_3_ solutions and subsequently reconstituted with water to transfer all inorganic components into sample solutions. Wet ashing in a concentrated solution of HNO_3_, or its combination with concentrated solutions of H_2_SO_4_ or HClO_4_, is also applied and realized in open- (Nanda et al. 2003; Rashed and Soltan 2004) or closed- (Madejczyk and Baralkiewicz 2008) vessel systems. Both digestion approaches certainly enable the decomposition of the carbohydrate-rich honey matrix and release simple ions through which nonspectral and spectral interferences affecting the sample solution introduction, in addition to dissociation and atomization processes in the air-acetylene flame, respectively, are eliminated (Uren et al. 1998; Lachman et al. 2007; dos Santos et al. 2008). Unfortunately, using this manner of sample preparation, many problems may be encountered during the analysis of honey by FAAS. Apparently, dry ashing is laborious, and it takes a long time to prepare suitable sample solutions before measurements. A risk of losses of trace elements (including Cu among others) due to volatilization is quite high and frequent with this method (Zukowska and Biziuk 2008). When using wet-ashing procedures, only small amounts of samples are commonly handled at once; hence, trace elements, such as Cu, might not be detected at sufficiently low levels.

Methods of direct and nondestructive analysis of honey as to the content of Cu and other environmentally, toxicologically, and nutritionally relevant trace elements, such as the simple dissolution of samples in water and measurements of resulting solutions or other faster and less laborious approaches, are quite uncommon (Hernandez et al. 2005; dos Santos et al. 2008). It seems reasonable to suppose that alternative approaches to drastic oxidative treatments with continued heating of honey samples would be both needed and desired to simplify and shorten the analysis using FAAS. With respect to this problem, a possibility of the direct determination of Cu in solutions of honey subjected to a solid-phase extraction (SPE)–based preconcentration/separation procedure was verified. To retain traces of Cu and the separate aforementioned monosaccharides, the sorption behavior of a gel-type strong acidic cation exchanger Dowex 50 W × 8-400 was examined. Then conditions of the two-step elution, first removing K and Na ions from honey’s resin beds and then recovering enriched Cu traces, was optimized. The analytical performance of the developed SPE preconcentration/separation procedure was evaluated and the method was applied to the analysis of freshly ripened monoflower and multiflower honeys as to their content of Cu.

## Materials and Methods

### Instrumentation

All measurements of element concentrations were performed with a Perkin Elmer (Waltham, MA, USA) single-beam atomic absorption spectrometer model 1100B. This instrument is equipped with a laminar-flow burner—including an end cup with a drain interlock assemblage, a flow spoiler, a burning mixing chamber, and a single-slot burner head—for a very sharp air-acetylene-lean flame. A stainless steel nebulizer was used to aspirate all solutions. Measurements of Cu concentrations were made in an absorption mode (FAAS); K and Na were measured using the instrument operated in an emission mode [flame optical emission spectrometry (FOES)]. Readings of absorbance and emission intensities were performed using time-average integration. In each read cycle, three readings were integrated at 0.1-second intervals during a 3-second integration time and averaged. All instrumental settings recommended by the manufacturer were used for measurements of Cu by FAAS and K and Na by FOES. Instrumental detection limits for Cu, K, and Na, based on 3 × SD of average (*n* = 5) intensities for a water blank, were 6.8, 0.4, and 1.1 μg/L, respectively. The precision of the determination of Cu, K, and Na concentrations, as relative SDs of average measurements (*n* = 3) of 0.05, 0.10, and 0.20 mg/L (Cu) and 0.02, 0.05 and 0.10 mg/L (K, Na), were in the range 1.7–6.3, 1.4–5.6 and 2.1–6.7 %, respectively.

A Thermo Scientific (Bremen, Germany) single-beam Spectronic 20D+ digital visible spectrophotometer was applied for determining the sum of concentrations of fructose and glucose by the Somogyi–Nelson (arseno-molybdate) method (Fournier 2005). To measure concentrations of both monosaccharides, 10 μL of analyzed sample solutions were incubated at 90 °C for 10 min with addition of 0.5 mL of a 6.0 g/L alkaline CuSO_4_ solution. After cooling, 1.0 mL of an arseno-molybdate reagent solution, which was obtained by mixing a 50 g/L (NH_4_)_6_Mo_7_O_24_ solution with a 6.0 g/L Na_2_HAsO_4_ solution in the presence of a 0.78 mol/L H_2_SO_4_ solution, was added to resulting reaction mixtures and vigorously shaken. After 10 more minutes, they were diluted to 10.0 mL and remixed. Finally, absorbances related to a polymolybdate complex of intensive blue color produced in these solutions were measured spectrophotometrically at 520 nm. Sums of concentrations of fructose and glucose were determined against an appropriate reagent blank and standard working solutions containing from 1 to 50 mg/L of glucose.

### Reagents and Materials

Highly pure water produced by a WIGO (Wroclaw, Poland) PRO-11G reverse osmosis water purification system was used throughout. Solutions of ACS grade concentrated reagents, i.e., 30 % (m/m) H_2_O_2_, 37 % (m/m) HCl, 65 % (m/m) HNO_3_, and 95–98 % (m/m) H_2_SO_4_, were supplied by J. T. Baker (Deventer, Netherlands). Other reagents—including d-fructose (purity > 98 %), d-glucose (purity > 98 %), CuSO_4_·5H_2_O, (NH_4_)_6_Mo_7_O_24_·4H_2_O, and Na_2_HAsO_4_·7H_2_O—were of analytical reagent grade and purchased from POCH (Gliwice, Poland). Merck KGaA (Darmstadt, Germany) Titrisol single-element standards for AAS containing 1,000 mg of Cu (as CuCl_2_ in water), K (as KCl in water), and Na (as NaCl in water) were used to prepare 1,000 mg/L single-element stock standard solutions of these elements.

Working standard solutions of Cu, K, and Na for calibration of the spectrometer and 100-mL working standard solutions containing 0.2, 20, and 5.0 mg/L of Cu, K, and Na with admixture of fructose and glucose at concentrations of 40 g/L were prepared by diluting appropriate bulk standard solutions and adding appropriate amounts of solid monosaccharides. The latter solutions were applied to investigate sorption and desorption conditions of the cation exchangers used for SPE. Their composition corresponded to typical concentrations of K and Na as well as predominating fructose and glucose in 10 % (m/v) water solutions of monofloral and multifloral Polish honeys (Madejczyk and Baralkiewicz 2008; Chudzinska and Baralkiewicz 2010). In addition, these solutions were adjusted to pH 3.5, 4.0, and 4.5, respectively, using a 0.010 mol/L HCl solution.

Calibration curves for FAAS (Cu) and FOES (K and Na) measurements were based on six standard solutions of Cu (0.05–1.00 mg/L), K (0.01–1.00 mg/L), and Na (0.01–1.00 mg/L). For the determination of K and Na, simple water standard solutions were only used. For the quantification of Cu, simple water standard solutions were used, and two additional sets of matrix matching standard solutions were prepared as well. The first set of standard solutions contained 40 g/L of fructose and glucose and was used for the evaluation of sorption properties of the resin. The second set of standard solutions contained adequate portions of HCl depending on the elution solution applied at the desorption step.

The strongly acidic cation exchange styrene–divinylbenzene resin Dowex 50 W × 8-400 with sulphonic acid functional groups (particle size 38–75 μm, H^+^ form) was provided by Sigma-Aldrich (Saint Louis, MO, USA). The resin was packed into Sigma-Aldrich glass columns (1.0-cm inner diameter) with glass coarse frits and polytetrafluoroethylene stopcocks. Flow rates of all solutions loaded onto SPE columns and passed through resin beds of Dowex 50 W × 8-400 were controlled using Cole-Parmer (Vernon Hill, Illinois, USA) four-channel MasterFlex L/S peristaltic pumps.

SPE columns were filled with slurries of water-cleaned 1.0-g portions of the resin suspended in water. Resin beds formed were flushed with 20 mL of water and then conditioned with 10 mL of a 2.0 mol/L HCl solution at 5.0 mL/min. Finally, columns were rinsed at the same flow rate with 50 mL of water to remove excess HCl.

### Honey Samples and Their Preparation

Ripened honeys—including acacia, heather, goldenrod, lime, multiflower and rape—were taken from an apiary located in suburbs of Wroclaw (Lower Silesian Province, Poland). Honeys were kept in a laboratory (at room temperature and in a dark place) in the original glass jars in which they were dispatched. Before sampling, contents of jars were vigorously stirred with a glass stirring bar to homogenize analyzed subsamples.

For wet ashing of honey samples, their representative 2.5-g portions were placed in 250-mL beakers covered with watch glasses, dissolved in 10 mL of water, and treated with 10 mL of a concentrated HNO_3_ solution. Samples were refluxed at 80–90 °C for approximately 2.5–3.0 h to avoid spattering and boiling. After cooling, remained digests (1–2 mL) were treated with 10 mL of a 30 % (m/m) H_2_O_2_ solution. Heating was prolonged for 1 h to decompose added H_2_O_2_ and decrease volumes of digested samples to approximately 1 to 2 mL. Resulting solutions were finally brought to 50 mL with water. The total time investment in this procedure was approximately 4 h.

**Fig. 1:**
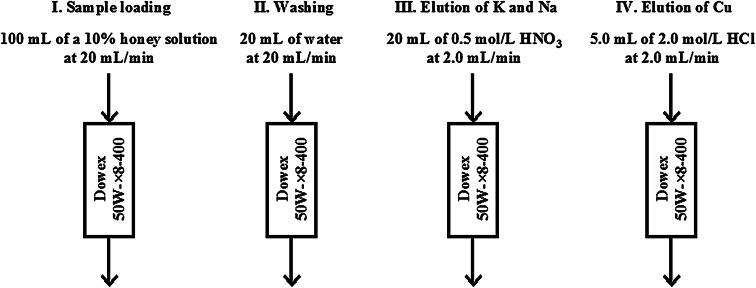
A schematic diagram of the SPE based preconcentration/separation procedure

For an SPE-based preconcentration/separation procedure with subsequent two-step elution, representative 10-g samples of analyzed honeys were placed in 250-mL beakers, dissolved at first in 10 mL of water, and then brought to 100 mL. Resulting 10 % (m/v) honey solutions were passed at 20 mL/min through SPE columns filled with 1.0 g of Dowex 50 W × 8-400 to retain Cu(II) ions and separate them from monosaccharides and anionic minerals. After sample loading, SPE columns were rinsed with 10 mL of water at 20 mL/min, and 20 mL of a 0.5 mol/L HNO_3_ solution was subsequently passed through them at 2.0 mL/min to remove matrix elements K and Na. At the end, 5.0 mL of a 2.0 mol/L HCl solution was used to elute Cu(II) before measurements of its concentrations by FAAS versus matrix matching standard solutions (see Fig. 1). The total time investment in this procedure was approximately three quarters of an hour including preparations of resin beds and sample solutions.

For both sample preparation procedures, analyses were made on three parallel samples. Respective procedural blanks were prepared and considered in final results given as average values with standard uncertainties.

## Results and Discussion

### Sorption Behavior of Dowex 50 W × 8-400

The sorption behavior of Cu(II), K(I), and Na(I) ions, in addition to that of fructose and glucose, was examined passing 100-mL working standard solutions (pH 3.5, 4.0, and 4.5) at 2.0 mL/min through SPE columns filled with the resin studied. 10 mL portions of the resulting effluents collected at the end of the solution loading were next analyzed by FAAS and FOES for the contents of Cu and K with Na, respectively, which were not retained by the resin. Matrix matching standard solutions containing 40 g/L of fructose and glucose were used for calibration in this case. Sums of concentrations of fructose and glucose were measured using the Somogyi–Nelson spectrophotometric method. Concentrations of Cu, K, and Na retained on resin beds under given conditions were evaluated by subtracting concentrations of these elements determined in portions of effluents collected from their initial concentrations in working standard solutions. Retention efficiencies for Cu, K, and Na (in  %) were assessed by relating concentrations of retained elements to their original concentrations in loaded working standard solutions. Separation efficiencies (in  %) for fructose and glucose were evaluated by relating concentrations of both monosaccharides found in aforementioned portions of effluents to their initial concentrations in loaded working standard solutions.

It was established that Dowex 50 W × 8-400 quantitatively retains Cu(II) ions from solutions containing fructose and glucose (Table 2). Apparently all effluents collected were found to contain Cu at concentrations lower than the respective detection limit (DL) of Cu obtained by FAAS. Fructose and glucose were established to pass through beds of the resin completely unretained. In respect to this, it seems that Cu can be adequately preconcentrated before determination of its concentrations by FAAS without matrix effects originating from fructose and glucose. Unfortunately, it was ascertained that the resin has a high capacity for K and Na ions as well, which present in working standard solutions at concentrations, respectively, 100 and 25 times greater than the concentration of Cu, were also quantitatively retained in the studied pH range of 3.5–4.5.

**Table 2:** Retention (for Cu, K and Na) and separation (for fructose with glucose) efficiencies (in %) achievable with Dowex 50 W × 8-400 at pH 3.5–4.5

PH	Cu	K	Na	Monosaccharides^b^
3.5	>97.0^a^	99.9 ± 0.1	100.0 ± 0.1	101.4 ± 1.8
4.0	>97.0^a^	99.9 ± 0.1	100.0 ± 0.1	99.1 ± 7.8
4.5	>97.0^a^	100.0 ± 0.1	100.0 ± 0.1	95.2 ± 6.1

Average values (*n* = 3) ± SDs

^a^Concentrations of Cu in effluents are lower than the respective DL

^b^Sum of concentrations of fructose and glucose

The influence of the flow rate with which working standard solutions were passed through SPE columns on the retention of elements and the separation of fructose and glucose was examined in the range from 2.0 to 20 mL/min. Selected working standard solutions (pH 4.0) were passed through SPE columns filled with the Dowex 50 W × 8-400 resin, whereas effluents were collected during the passage of these solutions through columns and analyzed to evaluate respective retention and separation efficiencies (average values, *n* = 3).

It was found that in all cases, concentrations of Cu determined using FAAS in all collected column effluents were found to be lower than the respective DL, whereas average concentrations of sum of glucose and fructose in these effluents contributed from 98.6 to 103.8 % to their original concentrations in working standard solutions loaded onto SPE columns. Based on these results, it was concluded that the Dowex 50 W × 8-400 resin maintains the quantitative retention of Cu(II) ions (>97.0 %) and achieves their complete separation from glucose and fructose regardless of the solution flow rate applied. K and Na ions were also established to be exhaustively retained by Dowex 50 W × 8-400 resin beds in these conditions. Retention efficiencies of K and Na for these resins were found to change correspondingly from 99.9 to 100.0 % (K) and from 99.0 to 99.9 % (Na).

### Desorption Behavior of Dowex 50 W × 8-400

Because the presence of a high concentration of K and Na may pose problems in FAAS determinations of Cu (Pinta 1975), it was expected that the selective elution of these elements (keeping Cu untouched on resin beds) from SPE columns would be the most favorable. In view of that, the usefulness of 0.2 and 0.5 mol/L HNO_3_ and HCl solutions used for washing SPE columns and removing K and Na ions from resin beds of Dowex 50 W × 8-400 was evaluated. At first, working standard solutions (pH 4.0) were driven through SPE columns at 20 mL/min. After that, four 10-mL portions of tested HNO_3_ or HCl solutions were passed at 2.0 mL/min through SPE columns to elute K and Na ions. Respective 10-mL portions of column eluates were collected and analyzed on the content of Cu, K, and Na using calibration with simple water standard solutions. Elution efficiencies (in  %) for Cu, K, and Na under these conditions were evaluated relating concentrations of these elements found in respective eluates to their original concentrations in loaded working standard solutions.

As can be seen from Table 3, the best conditions that uphold the total elution of K and Na ions from resin beds of Dowex 50 W × 8-400 and preserve Cu untouched on SPE columns were achieved with 20 mL of a 0.5 mol/L HNO_3_ solution. Using this solution, it was possible to elute 95.5 ± 3.7 % of total K and 97.9 ± 4.6 % of total Na retained on SPE columns, whereas Cu remained preserved. Concentrations of Cu in all eluates collected were determined to be lower than its DL obtained by FAAS.

**Table 3:** Elution efficiencies (in  %) of Cu, K, and Na from resin beds of the strong cation exchange resin Dowex 50 W × 8-400 obtained using 0.2 and 0.5 mol/L HNO_3_ and HCl solutions

Cu				K					Na				
I	II	III	IV	I	II	III	IV	Sum	I	II	III	IV	Sum
0.2 mol/L HNO_3_													
<0.3^a^	<0.3^a^	<0.3^a^	0.6 (0.3)	0.5 (0.3)	7.1 (3.6)	34.5 (5.7)	25.6 (6.0)	67.7 (9.0)	28.2 (5.8)	57.7 (5.0)	16.1 (9.1)	1.0 (0.6)	103.0 (11.9)
0.5 mol/L HNO_3_													
<0.3^a^	<0.3^a^	0.7 (0.3)	1.2 (0.4)	42.0 (1.3)	53.5 (3.5)	9.3 (4.5)	0.6 (0.2)	105.4 (5.9)	90.5 (1.6)	7.4 (4.3)	1.5 (1.1)	0.3 (0.1)	99.7 (4.7)
0.2 mol/L HCl													
<0.3^a^	0.4 (0.1)	0.8 (0.1)	1.2 (0.1)	0.1 (0.1)	1.1 (0.5)	5.0 (2.4)	24.4 (1.8)	30.6 (3.2)	1.7 (0.1)	72.6 (2.3)	23.7 (1.9)	0.5 (0.4)	98.5 (3.0)
0.5 mol/L HCl													
<0.3^a^	0.7 (0.3)	1.5 (0.1)	3.2 (0.4)	5.1 (0.4)	87.4 (4.1)	6.6 (1.7)	1.3 (0.5)	100.4 (4.5)	97.9 (6.0)	4.5 (0.9)	1.2 (0.1)	1.3 (0.8)	104.9 (6.1)

Four 10-mL portions (I through IV) of eluting solutions were passed through resin beds

Average values (*n* = 3) with SDs in parentheses

^a^Concentrations of Cu in effluents are lower than the respective DL

Finally, remaining Cu was stripped from SPE columns with 1.0, 2.0, 3.0, and 4.0 mol/L HCl solutions and respective elution efficiencies achieved with these solutions were assessed to select optimum desorption conditions. For that purpose, working standard solutions (pH 4.0) were passed at 20 mL/min through resin beds of Dowex 50 W × 8-400; next, K and Na ions were selectively eluted by passing through SPE columns 20 mL of a 0.5 mol/L HNO_3_ solution at 2.0 mL/min. Subsequently, 5.0 mL of a given HCl solution were passed at 2.0 mL/min through resin beds of Dowex 50 W × 8-400 to recover Cu. Adequate 5.0-mL portions of eluates were collected and concentrations of Cu were determined in them by FAAS against standard solutions containing corresponding concentrations of HCl.

It was found that, except for a 1.0 mol/L HCl solution, other HCl solutions provide quantitative recoveries (average values, *n* = 3) of Cu, *i.e.*, 101.3 ± 1.8 % in case of a 2.0 mol/L HCl solution, 100.6 ± 2.8 % in case of a 3.0 mol/L HCl solution, and 94.5 ± 3.7 % in case of a 4.0 mol/L HCl solution.

Considering all results obtained, it was decided that 20 mL of a 0.5 mol/L HNO_3_ solution and 5.0 mL of a 2.0 mol/L HCl solution would be used for the two-step elution in the developed SPE preconcentration/separation procedure. It is worth noticing that Dowex 50 W × 8-400 is an unspecific resin, and therefore other metal ions, *e.g.*, Ca(II), Fe(III), Mg(II), Mn(II), and Zn(II), are quantitatively retained and can be eluted along with Cu(II) ions (Pohl, Stecka and Jamroz 2012a, 2012b). Under these conditions, it is possible to preconcentrate all of these metals from the honey matrix in a corresponding SPE procedure and determine them by FAAS without any interference resulting from the presence of high amounts of fructose, glucose, K, and Na. In addition, as verified in both cited artilces, the measurements of concentrations of manganese and zinc in the final eluates achieved are free from matrix effects related to the presence of relatively high amounts of calcium and magnesium.

### Analytical Characteristics and Application

Due to lack of an adequate certified reference material for honey, the accuracy of the proposed SPE preconcentration/separation procedure, as well as the method of the honey analysis, was studied by recovery experiments and the analysis of 10 % (m/v) solutions of rape, goldenrod, and heather honeys spiked with known amounts of Cu(II) ions. Cu was added to these sample solutions at concentrations of 0.005, 0.02, 0.05, 0.10, and 0.20 mg/L, and this corresponded to 0.10, 0.20, 0.50, 1.00, and 2.00 μg/g of Cu in honey samples. Recoveries achieved (average values, *n* = 3) were found to be within 94.7– 97.1 % (at 0.10 μg/g), 93.6–98.8 % (at 0.20 μg/g), 97.3–o 101.2 % (at 0.50 μg/g), 96.9 % to 102.4 % (at 1.00 μg/g), and 97.8 % to 100.2 % (at 2.00 μg/g), respectively, which proves the good accuracy of this procedure. The method DL for Cu as assessed with FAAS and the SPE preconcentration/separation procedure was 3.6 ng/g. It referred to the absorbance of 3 × SD of mean measurements of respective procedural blanks, *i.e.*, 100-mL working standard solutions with 20 mg/L of K, 5.0 mg/L of Na, and 40 g/L of fructose and glucose.

To illustrate the suitability of the devised procedure, the SPE preconcentration/separation with the two-step elution was subjected to the analysis of six different raw monoflower and multiflower honeys. Results of this analysis along with results obtained after wet ashing of analyzed honeys are listed in Table 4. As can be seen, the most commonly used in the honey analysis wet-ashing procedure was found to be of low precision and detectability. Relative SDs obtained for the given data set were within 21–31 %. In addition, it was not possible to detect Cu in acacia and multiflower honeys. In the latter case, the DL of 8.1 μg/L of Cu was assessed with FAAS in solutions of digested honey samples, and this corresponded to 0.16 μg/g of Cu in the honey matrix.

**Table 4:** Concentrations (in μg/g) of Cu in raw honeys as determined by FAAS after wet ashing of samples with HNO_3_ and H_2_O_2_ (I) and dissolution of samples in water followed by SPE and two-step elution (II)

Honey	I	II	*C*-test^a^
Acacia	<0.16^b^	0.169 ± 0.010	NA
Goldenrod	0.444 ± 0.092	0.418 ± 0.014	+0.329
Heather	0.358 ± 0.088	0.274 ± 0.013	+1.335
Lime	0.330 ± 0.098	0.259 ± 0.02	+1.017
Multiflower	<0.16^b^	0.186 ± 0.019	NA
Rape	0.277 ± 0.087	0.325 ± 0.024	−0.752

*NA* not applied

Average values (*n* = 3) ± SDs

^a^Values of *C*-test calculated for compared mean concentrates obtained after wet ashing and SPE with two-step elution (*C*_critical_ of 4.303 at the 95 % level of significance)

^b^Method DL

By contrast, the proposed SPE preconcentration/separation procedure allowed measuring Cu concentrations with the precision (as RSD) in the range of 3–10 % as a result of the determination of 100 time greater concentrations of Cu compared with those in digested sample solutions. Indeed, according to the *F*-test applied at the 95 % level of significance, variances of mean concentrations of Cu in goldenrod, heather, lime, and rape honeys achieved with the wet-ashing procedure were significantly greater than those obtained with the proposed SPE preconcentration/separation procedure. For that reason, the significance of differences between mean concentrations of Cu resulted from using both sample preparation procedures was tested using Cochran and Cox test with the critical value (C_critical_) of 4.303 at the 95 % level of significance (Miller and Miller 2005). No difference between the compared two sets of results was found (C_calculated_ < C_critical_) suggesting that both sample preparation procedures provide statistically indistinguishable results, although the devised SPE preconcentration/separation procedure is approximately four times faster and results in much better precision.

In addition, it was verified that the acidification of prepared honey solutions, performed to dissociate eventual complexes of Cu with possible organic ligands and release simple Cu ions, was not necessary. Differences found between results obtained through the analysis of acidified (with HNO_3_ at 0.1 mol/L) and not acidified solutions of honeys were statistically insignificant according to Student *t* test performed at the 95 % level of significance. This could be explained by strong interactions of sulphonic functional groups of the strong acidic cation exchanger with possible complexes of Cu as described previously (Odegard and Lund 1997; Ozdemir and Gucer 1998) or even a mechanical retention of such organic complexes by the gel-type cation exchange resin used here.

## Conclusion

In this study, a sample preparation procedure enabling to enrich traces of Cu in honey samples and separate this environmentally and toxicologically relevant element from a bulk matrix of fructose and glucose, as well as interferences originating from the presence of K and Na, has been developed for the FAAS analysis. The proposed procedure combines SPE on a gel-type strongly acidic cation exchanger Dowex 50 W × 8-400 with two-step elution, in which a 0.5 mol/L HNO_3_ solution was used for the complete elution of K and Na, whereas a 2.0 mol/L HCl solution was applied to quantitatively recover Cu. This procedure has been established to be a faster and easier alternative to time-consuming and laborious wet ashing of honey samples. The total time investment of this honey pretreatment—including the preparation of resin beds and honey sample solutions, the subsequent resin-based preconcentration, and the sequential elution before FAAS measurements—is approximately three quarters of an hour and approximately 5 times shorter than that required for wet digestion. The use of reagents is also much lower. The method provides precise, accurate, and interference-free determinations of Cu at the level of 10^−1^ μg/g and could be quite useful for analysts and environmentalists dealing with honey. Using commercially available strong cation exchange sorbent cartridges and vacuum manifolds, it would be possible with the procedure developed to prepare 12–15 honey samples at once.
